# Genomic Subtypes of GISTs for Stratifying Patient Response to Sunitinib following Imatinib Resistance: A Pooled Analysis and Systematic Review

**DOI:** 10.1155/2018/1368617

**Published:** 2018-08-26

**Authors:** Siyuan Tan, Ping Chen, Jiafu Ji, Shanshan Guo, Dapeng Yu, Tetsuya Asakawa, Yu Zhou, Masanobu Abe, Liang Zong

**Affiliations:** ^1^Department of Gastrointestinal Surgery, Northern Jiangsu People's Hospital, Clinical Medical College, Yangzhou University, Yangzhou, Jiangsu Province, China; ^2^Department of Gastrointestinal Surgery, Key Laboratory of Carcinogenesis and Translational Research (Ministry of Education), Peking University Cancer Hospital & Institute, Beijing, China; ^3^Department of Oncology, Graduate School of Medicine, Dalian Medical University, Dalian, Liaoning, China; ^4^Department of Neurosurgery, Hamamatsu University School of Medicine, Handayama, Hamamatsu, Shizuoka, Japan; ^5^Department of General Surgery, Suzhou Municipal Hospital (North Campus), Suzhou, Jiangsu, China; ^6^Division for Health Service Promotion, University of Tokyo Hospital, 7-3-1 Hongo, Bunkyo-ku, Tokyo 113-0033, Japan; ^7^Department of Gastrointestinal Surgery, Graduate School of Medicine, University of Tokyo, 7-3-1 Hongo, Bunkyo-ku, Tokyo 113-0033, Japan

## Abstract

**Objectives:**

Sunitinib (a second-line chemotherapeutic agent that inhibits multiple kinases, including KIT and PDGFR) is widely used in imatinib-resistant patients with gastrointestinal stromal tumors (GISTs). However, diverse responses to sunitinib have been observed in the clinic. We aimed to evaluate whether the different GIST genotypes could be used to stratify patient response to sunitinib.

**Methods:**

We searched the PubMed, Embase, and Cochrane databases and included English-language literature published up to August 31, 2017. Inclusion criteria were GIST patients with KIT exon 9, KIT exon 11, or PDGFRA mutations and those without KIT/PDGFRA mutations (termed the wild-type genotype) who were receiving sunitinib within a clinical trial, and the efficacy evaluation was clinical benefit rate (CBR), median progression-free survival (PFS), and overall survival (OS). Odds ratios (ORs) for CBR and hazard ratios (HRs) for PFS and OS with 95% confidence intervals (CIs) in sunitinib-treated GIST patients with different genotypes were compared.

**Results:**

Seven studies totaling 531 patients were included. Patients with KIT mutations showed an improved CBR to sunitinib compared to those with PDGFRA mutations. In particular, those with the KIT exon 9 or 11 mutation showed improved CBR over those with PDGFRA mutation. Moreover, GIST patients with the KIT exon 9 mutation showed improved CBR over those with the KIT exon 11 mutation. Patients without KIT/PDGFRA mutations (wild-type genotype) showed better CBR than those with PDGFRA mutations.

**Conclusion:**

GIST genotypes may be useful for stratifying patient response to sunitinib after imatinib resistance.

## 1. Introduction

Gastrointestinal stromal tumors (GISTs), the most common mesenchymal tumor of the GI tract, mainly harbor a mutation of either the protooncogene KIT or the platelet-derived growth factor receptor alpha (PDGFRA) [1–3]. Around 80–90% of patients with GISTs express KIT mutations; primary KIT mutation sites include exon 9, exon 11, exon 13, and exon 17. And 5–8% of GISTs harbor PDGFRA mutations [4, 5]; the PDGFRA mutations in GISTs occur in exon 18, exon 12, and exon 14. The remaining GISTs, which are traditionally classified as wild-type (WT), mostly be classified into succinate dehydrogenase B (SDHB) negative (include SDH mutation (A/B/C/D)) and SDHB positive (BRAF V600E mutation, RAS mutations, NF1-related) [5].

Surgical resection is the standard treatment for localized GISTs. Adjuvant imatinib is used for high-risk GISTs. Imatinib is a selective inhibitor of tyrosine kinases, specifically KIT, PDGFRA, and ABL kinases. Unfortunately, approximately 14% of GISTs show primary resistance to imatinib [6]. The benefit of imatinib primarily depends on the GIST genotype [7]. Indeed, our previous meta-analysis demonstrated that, among all genotypes, GIST patients with the KIT exon 11 mutation were the most sensitive to imatinib [8]. In addition to primary resistance, secondary resistance to imatinib was found to occur in 40% of patients with GISTs within 2 years of treatment [9, 10]. Therefore, a new treatment strategy is required following failure of imatinib therapy.

In patients with imatinib-resistant or imatinib-intolerant GIST, sunitinib has shown promising benefit in phase I–III clinical trials and is now widely recognized as second-line therapy worldwide. It is a second-generation oral multitargeted tyrosine kinase inhibitor (TKI) that exerts its antitumorigenic effects by targeting several kinases, including KIT, PDGFRA, and the vascular endothelial growth factor receptor (VEGFR) [11–13]. In addition, previous studies have confirmed that several KIT and PDGFRA mutations are associated with the response of GIST to sunitinib, including the KIT exon 9, 11, 13, and 17 mutations and the PDGFRA exon 12, 14, and 18 mutations [14]. However, due to a limited sample size, the results were inconclusive [14–20]. Therefore, we conducted a meta-analysis to elucidate which GIST genotype is the most sensitive to sunitinib following imatinib resistance.

## 2. Methods

### 2.1. Search Strategy

We searched the PubMed, Embase, and Cochrane databases for English-language limited literature published up to August 31, 2017. The search terms were “gastrointestinal stromal tumor,” “gastrointestinal stromal neoplasm,” “gastrointestinal stromal tumour,” “GIST,” and “sunitinib”. Two reviewers (S.T. and D.Y.) independently screened all titles for the first-round selection to exclude duplicate articles. After that they screened abstracts, following exclusion criteria for the second-round selection. Of the remaining articles, both reviewers independently evaluated the full text, following inclusion criteria for the third-round selection. Discrepancies between the two reviewers were resolved via discussion with three senior authors (J.J., P.C., and L.Z.).

### 2.2. Exclusion Criteria

The following studies or data were excluded if they met the following criteria:

1. Case reports, reviews, and letters were excluded.
2. Studies lacking genotype information or without any information of CBR or PFS or OS were excluded.
3. They reported on a clinical trial of sunitinib and placebo for primary GIST patients.

### 2.3. Inclusion Criteria

Articles were selected for inclusion if they (1) estimated the efficacy of sunitinib in the treatment of imatinib-resistant or imatinib-intolerant GIST patients with different genotypes; (2) were clinical trial studies; (3) contained sufficient data for evaluating odds ratios (ORs) with 95% confidence interval (CI); and (4) provided survival curves for evaluating hazard ratios (HRs) with 95% confidence interval (CI).

### 2.4. Data Extraction

The following information was carefully extracted from the selected studies: first author's name; publication year; therapeutic regimen; total number of KIT mutation cases, KIT exon 9 cases, KIT exon 11 cases, WT cases, and PDGFRA cases; and the clinical benefit rate (CBR), defined as patients with complete response (CR) + partial response (PR) + stable disease (SD) according to the Response Evaluation Criteria in Solid Tumors (RECIST), which rely solely on tumor size, and maximum standardized uptake value (SUVmax) reduction in positron emission tomography (PET) [21], median progression-free survival (PFS), and overall survival (OS) after sunitinib treatment. There was no minimum number of patients required for including a study in our meta-analysis.

### 2.5. Statistical Analysis

ORs and associated 95% CIs were used to assess the treatment efficacy of sunitinib in imatinib-resistant or imatinib-intolerant GIST patients with different genotypes. A weighted average of the individual adjusted log HRs was used to summarize the association between genotypes and PFS or OS, with the weights inversely proportional to the variance of the log HR of each study. Heterogeneity was assessed using forest plots by performing the *χ*^2^ test (assessing the *P* value) and calculating the *I*^2^ statistic. If *I*^2^ ≤ 50% (*P* > 0.10), the studies were considered to show significant homogeneity, and the fixed-effects model (the Mantel-Haenszel method) was selected. Conversely, if *I*^2^ > 50% (*P* ≤ 0.10), the studies were considered to show significant heterogeneity. If the heterogeneous data could not be eliminated, the random-effects model (the DerSimonian and Laird method) was used. The pooled OR or HR was determined by the *Z*-test, and a *P* ≤ 0.05 was considered statistically significant. Potential publication bias was evaluated by the funnel plot: asymmetry of the funnel plot is indicative of publication bias. Trim and fill analysis, which can adjust for funnel plot asymmetry, was also used. All analyses were performed using the STATA version 13.0 (Stata Corporation, College Station, TX, USA).

## 3. Results

### 3.1. Study Selection and Characteristics

**Figure 1:**
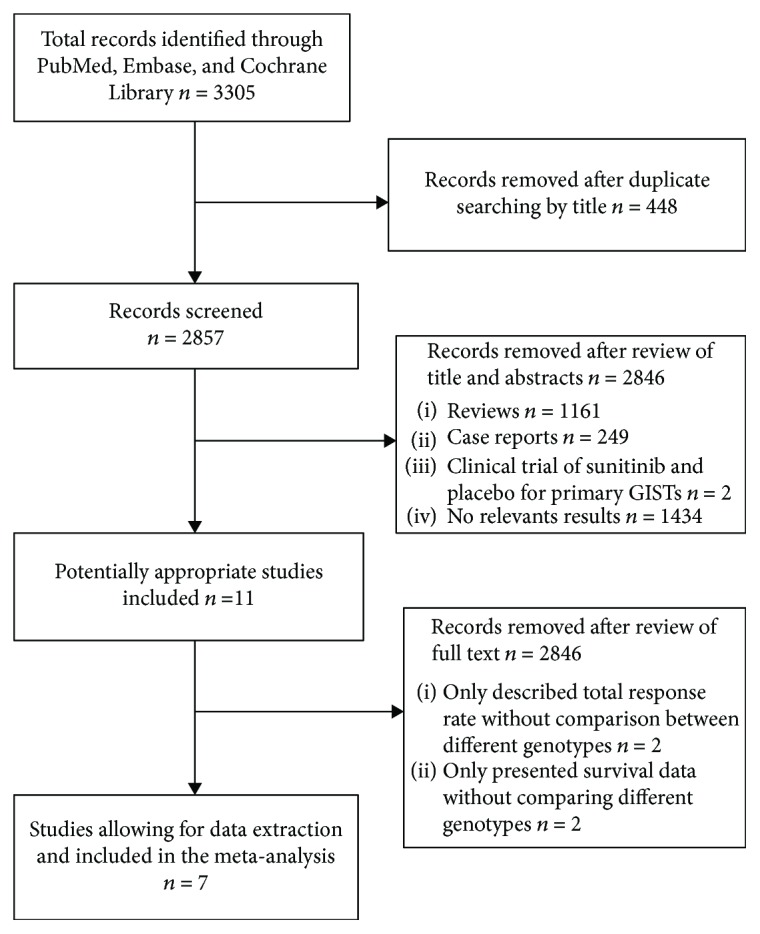


The flow diagram shows the study selection process used in this analysis (Figure 1). The studies by Matsumoto et al. [22] and Kefeli et al. [23] were excluded as they described the CBR without comparison between the different GIST genotypes [22, 23]. Similarly, the studies by Komatsu et al. [24] and Reichardt et al. [25] were excluded as they only presented survival data without comparing the different GIST genotypes. Finally, seven studies including 531 patients were used in the pooled analyses [14–20]. Almost all patients with GIST were diagnosed by histology and immunohistochemistry, and the polymerase chain reaction technique was used to determine the GIST genotype (mutational status). All patients were confirmed as imatinib-resistant or imatinib-intolerant. Tables 1–3 list the main characteristics of the included studies.

### 3.2. Results of the Meta-Analysis

#### 3.2.1. Comparison of Clinical Benefit Rate between Different GIST Genotypes

**Figure 2:**
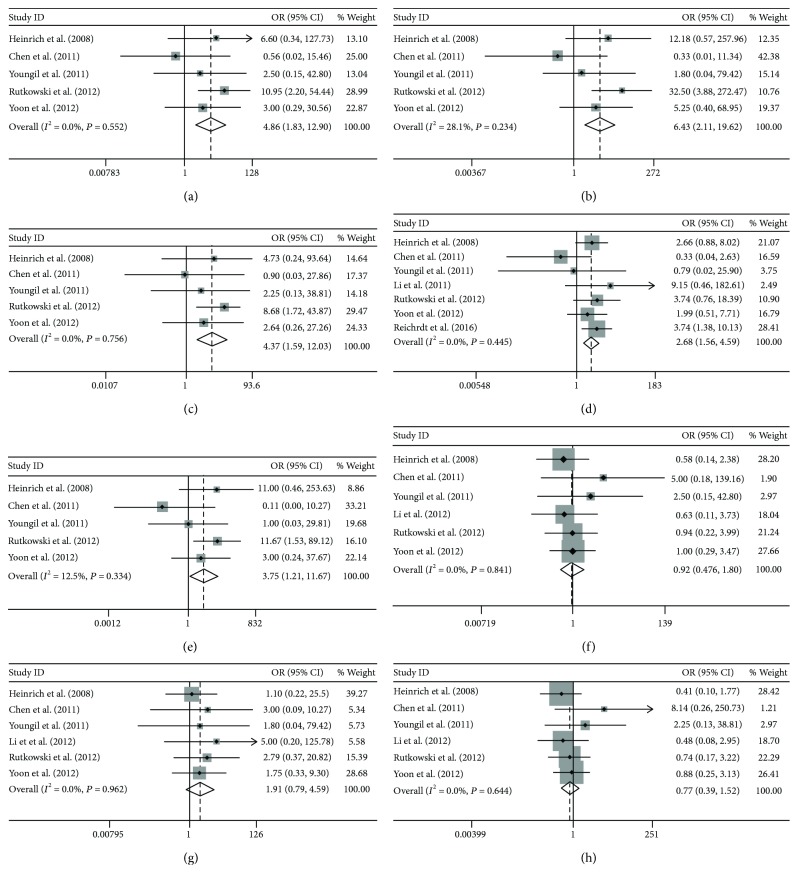


Statistically significant improvements in the CBR were observed in the KIT group versus the PDGFRA group (OR = 4.86, 95% CI: 1.83–12.90; *P* = 0.001, *P*_heterogeneity_ = 0.55), in the KIT exon 9 group versus the PDGFRA group (OR = 6.43, 95% CI: 2.11–19.62; *P* = 0.001, *P*_heterogeneity_ = 0.23), in the KIT exon 11 group versus the PDGFRA group (OR = 4.37, 95% CI: 1.59–12.03; *P* = 0.004, *P*_heterogeneity_ = 0.76), in the KIT exon 9 group versus the KIT exon 11 group (OR = 2.68, 95% CI: 1.56–4.59; *P* < 0.001, *P*_heterogeneity_ = 0.45), and in the WT group versus the PDGFRA group (OR = 3.75, 95% CI: 1.21–11.67; *P* = 0.022, *P*_heterogeneity_ = 0.33) (Figures 2(a)–2(e)). However, no significant differences were found between the KIT group and the WT group (OR = 0.92, 95% CI: 0.47–1.80; *P* = 0.82, *P*_heterogeneity_ = 0.84), the KIT exon 9 group and the WT group (OR = 1.91, 95% CI: 0.79–4.59; *P* = 0.15, *P*_heterogeneity_ = 0.96), or the KIT exon 11 group and the WT group (OR = 0.77, 95% CI: 0.39–1.52; *P* = 0.45, *P*_heterogeneity_ = 0.64) (Figures 2(f)–2(h)).

#### 3.2.2. Comparison of Progression-Free Survival and Overall Survival between Different GIST Genotypes

**Figure 3:**
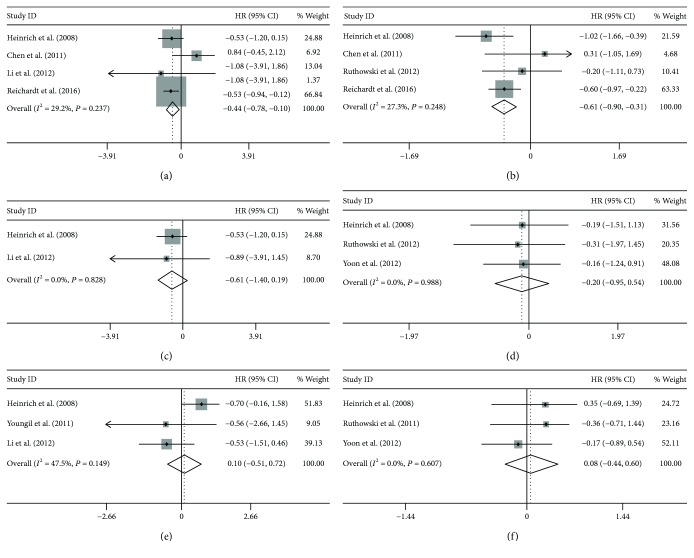


Only the KIT exon 9, KIT exon 11, and WT genotypes were assessed regarding PFS and OS due to the lack of data for GIST patients with PDGFRA genotypes. There were statistically significant longer PFS and OS in the KIT exon 9 group than the KIT exon 11 group (HR = −0.44, 95% CI: −0.78, −0.10; *P* < 0.01, *P*_heterogeneity_ = 0.24) (HR = −0.61, 95% CI: −0.90, −0.31; *P* < 0.001, *P*_heterogeneity_ = 0.25) (Figures 3(a) and 3(b)). Intriguingly, there were no statistical differences in PFS and OS between the KIT exon 9 group and the WT group (HR = −0.61, 95% CI: −1.40, 0.19; *P* = 0.13, *P*_heterogeneity_ = 0.83) (HR = −0.20, 95% CI: −0.95, 0.54; *P* = 0.60, *P*_heterogeneity_ = 0.99) (Figures 3(c) and 3(d)), or the KIT exon 11 group and the WT group (HR = 0.10, 95% CI: −0.51, 0.72; *P* = 0.74, *P*_heterogeneity_ = 0.15) (HR = 0.08, 95% CI: −0.44, 0.60; *P* = 0.77, *P*_heterogeneity_ = 0.61) (Figures 3(e) and 3(f)).

### 3.3. Publication Bias

**Figure 4:**
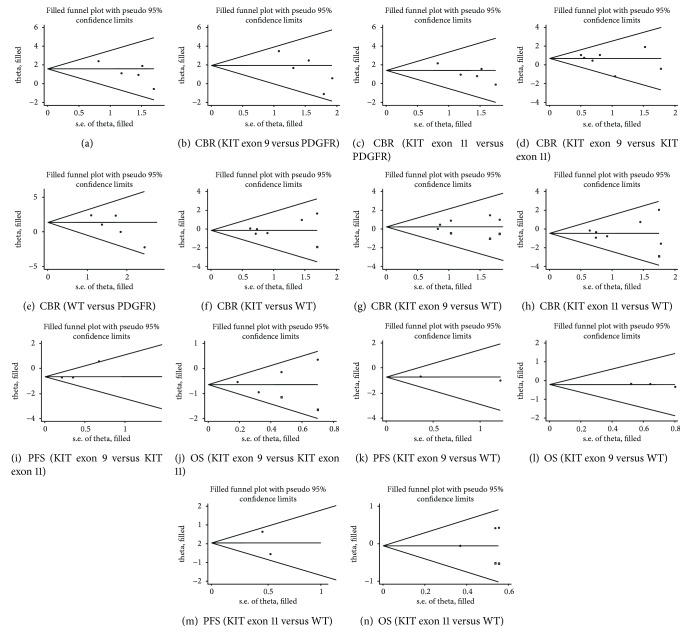


After the trim and fill analysis, we used funnel plots to evaluate the publication bias of each report. However, no obvious asymmetry was observed, indicating a lack of publication bias (Figures 4(a)–4(n)).

## 4. Discussion

Several mutations have been confirmed to be related to the prognosis of GIST, including KIT mutations in exons 9, 11, 13, and 17 and PDGFRA mutations in exons 12, 14, and 18 [26]. In this study, we conducted a pooled analysis to evaluate whether these genomic subtypes of GISTs could also be used to stratify patient response to sunitinib after failure of imatinib treatment. Our analysis indicates that KIT exon 9-mutant GISTs show the best response to sunitinib, followed by WT and KIT exon 11-mutant genotypes, while GIST patients with the PDGFRA mutations are least responsive to sunitinib after imatinib resistance.

The CBR from sunitinib (defined as patients with CR + PR + SD) in all GIST patients with any genotype was evaluated with computed tomography according to RECIST [21]. Our meta-analysis showed that CBR was significantly improved in GIST patients harboring KIT mutations compared to those with PDGFRA mutations. Improved CBR was also observed in GIST patients specifically with KIT exon 9/11 mutations compared to those with PDGFRA mutations. GIST patients with the WT genotype also showed better CBR than those with PDGFRA mutations. Meanwhile, GIST patients with KIT exon 9/11 mutations showed no difference in CBR to those with WT genotype. However, GIST patients with the KIT exon 9 genotype showed improved CBR compared to those with KIT exon 11 genotype, suggesting the treatment response in the WT genotype is somewhere between the KIT 9-mutant and the KIT 11-mutant genotypes. Our analysis demonstrates that GIST patients with the KIT exon 9 mutation have the best CBR to sunitinib, and those with the PDGFRA mutation display the worst treatment outcomes.

We also examined PFS and OS among GIST patients with different genotypes. Due to the lack of data for GIST patients with PDGFRA genotypes, we only compared data for patients with the KIT exon 9 mutation, the KIT exon 11 mutation, and the WT. We extracted the survival data from each study [27, 28] and computed the HR along with the 95% CI. The result of pooled HR with 95% CI showed that PFS and OS were prolonged in sunitinib-treated GIST patients with the KIT exon 9 mutation compared with the KIT exon 11 mutation while those with WT showed no difference with KIT exon 9 or 11. To explore the potential mechanism why different subtypes of GIST patients showed differential CBR, PFS, or OS to sunitinib, we suppose that this is probably due to the differential mutational sites that have different structural effects on receptor tyrosine kinases and have different consequences for interaction with sunitinib.

The dosage of sunitinib used in the studies included in this meta-analysis was 50 mg/day on a 4-weeks-on, 2-weeks-off schedule or 37.5 mg on a continuous daily dosing schedule. According to the study by George et al., continuous daily dosing appears to be a safe dosing strategy offering more stable effectiveness [29]. While these two dosages of sunitinib do not seem to impact the CBR and PFS of GIST patients overall [11, 17], we failed to find data comparing the CBR and PFS in patients with different GIST genotypes with respect to sunitinib dosage. Therefore, further research is warranted regarding sunitinib dose and patient response when stratified by GIST genotype. However, we suppose that GIST patients can be stratified to sunitinib treatment by molecular features, and relative low dose is recommended to sensitive subtypes.

Regardless of the limited number and small size of included studies, still many confounding factors such as different doses, varying treatment schedules, sources of patient, publication bias, and ethnicity might prevent us from reaching a more concise conclusion. A significant weakness of this study is the lack of integrate data of PFS and OS to assess the long-term effect of genotypes for GISTs treated with sunitinib. To overcome these limitations, a clinical trial with increased patient size is needed in the future.

## 5. Conclusion

This meta-analysis of the recent literature indicates that, among all known genotypes, patients with GISTs harboring the KIT exon 9 mutation are most likely to benefit from sunitinib treatment. Alternatively, sunitinib appears to be least effective in GIST patients with the PDGFRA genotype. Further research is required incorporating larger sample sizes of GIST patients with the KIT, WT, and PDGFRA mutations, a longer follow-up, and different dosages of sunitinib or preimatinib in diverse GIST genotypes. These studies will enable us to determine the best practice for sunitinib usage in the treatment of GIST. According to our meta-analysis results, we advise using sunitinib in the treatment of imatinib-resistant or imatinib-intolerant GIST patients, especially the KIT exon 9 mutation genotype, thus can significantly improve prognosis of GIST patients.

## Figures and Tables

**Table 1:** Genomic subtypes of GIST patients included in the meta-analysis.

Author (year)	Dosage	CBR (%)	Genotype (*n*/*N*)						
			KIT	Exon 9	Exon 11	Exon 13	Exon 17	WT	PDGFRA
Heinrich et al. (2008) [7]	50 mg/day (4/2) or 37.5 mg/day CDD	42	27/64	11/19	15/44	1/1	NA	5/9	0/4
Chen et al. (2011) [15]		62	12/19	3/6	9/12	0/1	NA	0/1	1/1
Koh et al. (2011) [16]		78	10/12	1/1	9/11	NA	NA	2/3	2/3
Li et al. (2012) [17]		68	19/29	6/6	13/22	NA	0/1	6/8	NA
Rutkowski et al. (2012) [18]		62	46/67	13/15	33/52	NA	NA	7/10	2/12
Yoon et al. (2012) [19]		49	29/58	7/11	22/47	NA	NA	6/12	1/4
Reichardt et al. (2016) [20]		67	NA/196	37/42	95/143	NA/5	NA/6	NA/9	NA/18

4/2: 4-weeks-on, 2-weeks-off; CDD: continuous daily dose; NA: not available; CBR: clinical benefit rate; *n* = number of patients with complete response (CR) + partial response (PR) + stable disease (SD) according to the Response Evaluation Criteria in Solid Tumors (RECIST); *N* = total number of patients with genotype; WT: wild-type.

**Table 2:** Hazard ratios (HRs) with 95% confidence intervals (CIs) for progression-free survival (PFS) and overall survival (OS).

Author (year)	PFS HR (95% CI)			OS HR (95% CI)		
	KIT exon 9 versus KIT exon 11	KIT exon 9 versus WT	KIT exon 11 versus WT	KIT exon 9 versus KIT exon 11	KIT exon 9 versus WT	KIT exon 11 versus WT
Heinrich et al. (2008) [7]	0.59 (0.30–1.16)	0.56 (0.24–1.26)	2.01 (0.85–4.74)	0.36 (0.19–0.68)	0.83 (0.22–3.11)	1.42 (0.50–4.01)
Chen et al. (2011) [15]	2.31 (0.64–8.32)	NA	NA	1.37 (0.35–5.42)	NA	NA
Koh et al. (2011) [16]	NA	NA	0.57 (0.07–4.28)	NA	NA	NA
Li et al. (2012) [17]	0.34 (0.02–6.42)	0.41 (0.02–4.31)	0.59 (0.22–1.59)	NA	NA	NA
Rutkowski et al. (2012) [18]	NA	NA	NA	0.82 (0.33–2.07)	0.73 (0.14–3.79)	1.43 (0.49–4.21)
Yoon et al. (2012) [19]	NA	NA	NA	1.55 (0.74–3.24)	0.85 (0.29–2.48)	0.84 (0.41–1.72)
Reichardt et al. (2016) [20]	0.59 (0.39–0.89)	NA	NA	0.55 (0.38–0.80)	NA	NA

NA: not available.

**Table 3:** Baseline characteristics of GIST patients included in the meta-analysis.

Author (year)	Gender		Age (year)		ECOG PS			Primary location			
	Male	Female	Median	Range	0	1	≥2	Stomach	Small bowel	Large bowel	Other
Heinrich et al. (2008) [7]	53	25	55	26–76	38	24	6	NA	NA	NA	NA
Chen et al. (2011) [15]	16	7	59	24–83	NA	NA	NA	8	11	3	1
Koh et al. (2011) [16]	12	10	55	29–75	NA	NA	NA	10	11	0	1
Li et al. (2012) [17]	40	15	NA	NA	5	31	19	16	25	6	8
Rutkowski et al. (2012) [18]	74	63	55	15–82	48	72	17	46	79	4	8
Yoon et al. (2012) [19]	55	33	59	25–76	72		16	29	47	5	7
Reichardt et al. (2016) [20]	139	91	60	11–83	87	114	27	NA	NA	NA	NA

NA: not available; ECOG: Eastern Cooperative Oncology Group performance status; Other: peritoneum/abdominal cavity/mesentery/omentum.
